# Quantifying changes in the bacterial thiol redox proteome during host-pathogen interaction

**DOI:** 10.1016/j.redox.2018.101087

**Published:** 2018-12-19

**Authors:** Kaibo Xie, Christina Bunse, Katrin Marcus, Lars I. Leichert

**Affiliations:** a Ruhr University Bochum, Institute of Biochemistry and Pathobiochemistry, Microbial Biochemistry, 44780 Bochum, Germany; b Ruhr University Bochum, Medizinisches Proteom-Center, 44801 Bochum, Germany

## Abstract

Phagocyte-derived production of a complex mixture of different oxidants is a major mechanism of the host defense against microbial intruders. On the protein level, a major target of these oxidants is the thiol group of the amino acid cysteine in proteins. Oxidation of thiol groups is a widespread regulatory post-translational protein modification. It is used by bacteria to respond to and to overcome oxidative stress. Numerous redox proteomic studies have shown that protein thiols in bacteria, such as *Escherichia coli* react towards a number of oxidants in specific ways. However, our knowledge about protein thiols in bacteria exposed to the complex mixture of oxidants encountered in the phagolysosome is still limited. In this study, we used a quantitative redox proteomic method (OxICAT) to assess the in vivo thiol oxidation status of phagocytized *E. coli*. The majority (65.5%) of identified proteins harbored thiols that were significantly oxidized (> 30%) upon phagocytosis. A substantial number of these proteins are from major metabolic pathways or are involved in cell detoxification and stress response, suggesting a systemic breakdown of the bacterial cysteine proteome in phagocytized bacteria. 16 of the oxidized proteins provide *E. coli* with a significant growth advantage in the presence of H_2_O_2_, when compared to deletion mutants lacking these proteins, and 11 were shown to be essential under these conditions.

## Graphical abstract

**Figure:**
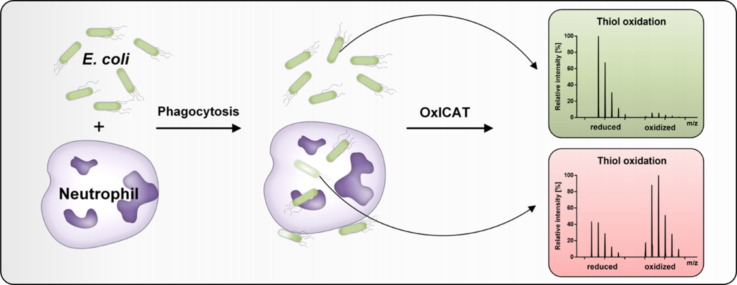

## 1 Introduction

Neutrophils are key players of the innate immune response. In response to invading microorganisms, they are recruited to sites of infection, where they internalize pathogens into compartments called phagosomes. During the process of phagocytosis, the NADPH oxidase 2 complex (NOX2) is assembled and activated [56]. This activation of NOX2 is dependent on the phosphorylation of its subunits, which is stimulated upon phagocytosis [4], [15]. As a result, superoxide anion (O_2_^•-^) is generated by one-electron reduction of phagosomal oxygen at the expense of cytosolic NADPH [53]. To compensate for the directional transport of electrons (e^-^) into the phagosome, protons (H^+^) are transported by voltage-gated H^+^-channels leading to acidification of the phagosomal compartment [17]. The superoxide anion O_2_^•-^ can disproportionate into hydrogen peroxide (H_2_O_2_), a reaction catalyzed by superoxide dismutase. H_2_O_2_ can, in turn, generate ^•^OH and hypochlorous acid (HOCl). The former is generated typically through Fenton-chemistry, the latter is produced catalytically by myeloperoxidase (MPO) [44], [58]. Such naturally occurring chemically reactive oxidants containing the element oxygen are often called “reactive oxygen species” (ROS).

In addition to those “ROS”, the phagosome also produces “reactive nitrogen species” (RNS), reactive oxidants containing the element nitrogen. Nitric oxide (^•^NO) is formed by the inducible nitric oxide synthase (iNOS) [64]. ^•^NO can further react with O_2_^•-^, generating peroxynitrite (ONOO-) and nitrogen dioxide (^•^NO_2_). Working together, these highly reactive oxygen and nitrogen species are crucial for the effective clearance of pathogenic intruders. Mice, which lack both iNOS and NOX2 and thus can produce neither ^•^NO nor O_2_^•-^ are therefore heavily compromised in their defense against bacteria [60].

Oxidants that are produced in the phagosome can react with and damage major cellular components of pathogens, including DNA, lipids and proteins. However, bacteria have their own mechanisms to protect themselves from oxidative stress. Antioxidant enzymes such as superoxide dismutases, catalases and peroxidases are some of the most common enzymes used by bacteria for detoxification and thus maintain the cell integrity [8].

In addition to those detoxifying enzymes, a few bacteria have evolved more specialized strategies to survive phagocytes including the pathogens *Mycobacterium tuberculosis* and *Salmonella enterica* serovar Typhimurium. Mechanisms involved in *M. tuberculosis* survival within the host cell include the inhibition of phagosome acidification and inhibition of the phagosome-lysosome fusion [43]. In comparison, intracellular *S.* Typhimurium translocate effector proteins into the host cell cytosol, alter the vesicular trafficking and modify the phagosome to their own advantage and survive in an acidified vacuole known as the “*Salmonella*-Containing Vacuole” (SCV). The biogenesis of SCV has been shown to be dependent on a type III secretion systems (T3SS) [45], [62]. A specific set of T3SS effectors has been shown to be directly involved in oxidative stress evasion strategies [27].

However, most bacteria are rapidly killed, once caught in the phagosome of neutrophils [69]. The mechanism, by which host-derived oxidants kill bacteria, is still not fully understood. On the protein level, a major target of oxidants is the thiol group of the amino acid cysteine. As is well known, the cysteine residue is used to keep conformational rigidity of structural proteins via the formation of disulfide bonds. However, within the cytosol of a cell, biological pathways often require catalytically active cysteines [52]. During the last few years, an increasing number of proteins involved in cellular stress response have been identified that are functionally regulated by reversible thiol oxidation including chaperones and transcription factors [21], [32], [71]. As shown by numerous studies, protein thiols in bacteria react towards a number of oxidants in a specific way [5], [37], [39]. However, the thiol redox proteome in bacteria that have encountered the complex mixture of phagosomal oxidants has not yet been investigated.

In this study, we established a method to separate phagocytized *E. coli* from extracellular *E. coli* after coincubation with the PLB-985 neutrophil-like cell line. Using a thiol trapping technique termed OxICAT, we then quantified the redox proteome of both the intracellular and extracellular *E. coli*. When compared to *E. coli* that were outside of the neutrophils and thus did not experience phagocytosis, 65.5% of the proteins identified in phagocytized *E. coli* showed an increase in cysteine oxidation of greater than 30%. The oxidized proteins were part of protein, nucleotide and carbohydrate metabolic pathways but were also involved in cell detoxification and stress response, which indicate a systemic oxidation of protein thiols. This suggests a total break-down of *E. coli*´s thiol proteome after encountering neutrophil phagocytosis. Moreover, as revealed by subsequent growth rate assays, 16 mutants, which lack proteins identified in our redox proteomic experiments, showed increased sensitivity towards oxidative stress. 11 of the genes encoding those proteins were essential for the growth of *E. coli* under otherwise sublethal oxidative stress conditions.

## 2 Experimental procedures

### 2.1 PLB-985 cell culture and differentiation

The human myeloid leukemia cell line PLB-985 (DSMZ, German collection of microorganisms and cell culture) was cultured in RPMI 1640 medium supplemented with 10% heat-inactivated fetal bovine serum (FBS), 1% GlutaMAX (Life Technologies, Darmstadt, Germany) at 37 °C, in a humidified atmosphere of 5% CO_2_ and passaged twice weekly. For granulocytic differentiation of cells, exponentially growing cells at a density of 2 × 10^5^/ml were cultured in RPMI 1640 medium supplemented with 10% FCS, 1% GlutaMAX and 1.25% DMSO for five days. On day four, cells were stimulated with 2000 U/ml interferon-γ (IFN-γ) [18], [51].

### 2.2 Phagocytosis of bacteria by PLB-985 cells

A culture of *E. coli* AM39 harbouring a generated vector containing roGFP2-Orp1 (for bacterial strains used in this study see Table 1) was grown to an OD_600_ of 0.4 at 37 °C with 100 µg/ml ampicillin. 100 µM IPTG was added to allow roGFP2-Orp1 expression overnight at 20 °C. The bacterial cells were washed twice in PBS (pH 7.4) and opsonized with 5 mg/ml human immunoglobulin G (hIgG, Sigma-Aldrich, St. Louis, MO) for 30 min at 37 °C. Afterwards, bacteria were washed twice with PBS and resuspended in PBS supplemented with 0.5% FBS to an OD_600_ of 0.1 (10^8^ cells/ml), unless described differently. Differentiated PLB-985 cells were washed once with PBS, resuspended in PBS supplemented with 0.5% FBS to a concentration of 10^7^ cells/ml and mixed with opsonized *E. coli* in the same volume (multiplicity of infection, MOI = 10). The cell suspension consisting of PLB-cells and *E. coli* was coincubated at 37 °C for 2 h.

**Table 1:** Bacterial strains used in this study.

**Strain**	**Genotype**	**Source or reference**
*E. coli AM39*	*E. coli* MG1655 (K−12 F^-^ λ^-^*ilvG*^*-*^*rfb−50 rph−1*) transformed with the roGFP2-Orp1 containing plasmid pCC_roGFP2-orp1	[14]^a^
*E. coli BW25113*	*lacI*^+^*rrnB*_T14_ Δ*lacZ*_WJ16_*hsdR*514 Δ*araBAD*_AH33_ Δ*rhaBAD*_LD78_*rph−1*	The National BioResource Project (National Institute of Genomics, Japan)^b^
	Δ(araB–D)567 Δ(rhaD–B)568	
	ΔlacZ4787(::rrnB−3) hsdR514 rph−1	
*E. coli JW3975*	*E. coli BW25113 ΔaceA*	See above^b^
*E. coli JW3974*	*E. coli BW25113 ΔaceB*	See above^b^
*E. coli JW2293*	*E. coli BW25113 ΔackA*	See above^b^
*E. coli JW0911*	*E. coli BW25113 ΔaspC*	See above^b^
*E. coli JW1737*	*E. coli BW25113 ΔastC*	See above^b^
*E. coli JW3712*	*E. coli BW25113 ΔatpA*	See above^b^
*E. coli JW5702*	*E. coli BW25113 Δcrp*	See above^b^
*E. coli JW4346*	*E. coli BW25113 ΔdeoB*	See above^b^
*E. coli JW2077*	*E. coli BW25113 ΔgatB*	See above^b^
*E. coli JW2873*	*E. coli BW25113 Δgcvt*	See above^b^
*E. coli JW3389*	*E. coli BW25113 ΔglpD*	See above^b^
*E. coli JW3897*	*E. coli BW25113 Δglpk*	See above^b^
*E. coli JW0710*	*E. coli BW25113 ΔgltA*	See above^b^
*E. coli JW3179*	*E. coli BW25113 ΔgltB*	See above^b^
*E. coli JW2011*	*E. coli BW25113 Δgnd*	See above^b^
*E. coli JW4103*	*E. coli BW25113 ΔgroL*	See above^b^
*E. coli JW5401*	*E. coli BW25113 ΔguaB*	See above^b^
*E. coli JW1122*	*E. coli BW25113 Δicd*	See above^b^
*E. coli JW3747*	*E. coli BW25113 ΔilvC*	See above^b^
*E. coli JW3592*	*E. coli BW25113 Δkbl*	See above^b^
*E. coli JW0336*	*E. coli BW25113 ΔlacI*	See above^b^
*E. coli JW0112*	*E. coli BW25113 Δlpd*	See above^b^
*E. coli JW0872*	*E. coli BW25113 Δlrp*	See above^b^
*E. coli JW2662*	*E. coli BW25113 ΔluxS*	See above^b^
*E. coli JW2447*	*E. coli BW25113 ΔmaeB*	See above^b^
*E. coli JW3205*	*E. coli BW25113 Δmdh*	See above^b^
*E. coli JW3194*	*E. coli BW25113 ΔnanA*	See above^b^
*E. coli JW2502*	*E. coli BW25113* Δ*ndk*	See above^b^
*E. coli JW2279*	*E. coli BW25113* Δ*nuoF*	See above^b^
*E. coli JW3933*	*E. coli BW25113* Δ*oxyR*	See above^b^
*E. coli JW5300*	*E. coli BW25113 ΔproQ*	See above^b^
*E. coli JW0325*	*E. coli BW25113 ΔprpD*	See above^b^
*E. coli JW2409*	*E. coli BW25113 ΔptsI*	See above^b^
*E. coli JW1843*	*E. coli BW25113 ΔpykA*	See above^b^
*E. coli JW4205*	*E. coli BW25113 Δpyrl*	See above^b^
*E. coli JW3907*	*E. coli BW25113 ΔrpmE*	See above^b^
*E. coli JW3037*	*E. coli BW25113 ΔrpsU*	See above^b^
*E. coli JW0713*	*E. coli BW25113 ΔsdhA*	See above^b^
*E. coli JW0714*	*E. coli BW25113 ΔsdhB*	See above^b^
*E. coli JW0717*	*E. coli BW25113 ΔsucC*	See above^b^
*E. coli JW0718*	*E. coli BW25113 ΔsucD*	See above^b^
*E. coli JW0396*	*E. coli BW25113 Δtgt*	See above^b^
*E. coli JW5478*	*E. coli BW25113 ΔtktA*	See above^b^
*E. coli JW3686*	*E. coli BW25113 ΔtnaA*	See above^b^
*E. coli JW1317*	*E. coli BW25113 Δtpx*	See above^b^
*E. coli JW1254*	*E. coli BW25113 ΔtrpC*	See above^b^
*E. coli JW3943*	*E. coli BW25113 ΔtufB*	See above^b^
*E. coli JW5394*	*E. coli BW25113 ΔucpA*	See above^b^
*E. coli JW1370*	*E. coli BW25113 ΔuspF*	See above^b^
*E. coli JW3063*	*E. coli BW25113 ΔuxaC*	See above^b^
*E. coli JW5126*	*E. coli BW25113 ΔycbX*	See above^b^
*E. coli JW1194*	*E. coli BW25113 ΔychF*	See above^b^
*E. coli JW1772*	*E. coli BW25113 ΔyeaG*	See above^b^
*E. coli JW2518*	*E. coli BW25113 ΔyfhR*	See above^b^
*E. coli JW2568*	*E. coli BW25113 ΔyfiQ*	See above^b^
*E. coli JW2647*	*E. coli BW25113 ΔygaM*	See above^b^
*E. coli JW2647*	*E. coli BW25113 ΔygaM*	See above^b^
*E. coli JW3040*	*E. coli BW25113 Δygjf*	See above^b^

a Degrossoli et al. [14].

b Baba et al. [2].

### 2.3 Real-time analysis of roGFP2-Orp1 oxidation state in *E. coli*

The measurement of roGFP2-Orp1 oxidation during the coincubation with PLB-985 cells was done in a 96-well format as described previously [14]. In short, 50 µl of *E. coli* expressing roGFP2-Orp1 at a final OD_600_ of 0.1 were either mixed with 50 µl of PLB-985 cells at a final concentration of 10^7^ cells/ml or with the respective reagents in a 96-well plate (Nunc black, clear-bottom, Rochester, NY). The fluorescence intensity was measured every minute for 2 h at the excitation wavelength 405 nm and 488 nm and the emission wavelength 510 nm. The 405/488 nm ratio was calculated using Excel 2016 (Microsoft, Redmond, WA) and visualized using GraphPad Prism (version 6.01, GraphPad, San Diego, CA).

### 2.4 Fractionation of phagocytized and non-phagocytized bacteria

Differentiated PLB-985 cells were co-cultivated with bacteria as described above for 2 h. Afterwards, the PLB cells were washed twice with ice-cold PBS in order to remove extracellular bacteria (500 g, 5 min, 4 °C), the resulting supernatant containing non-phagocytized bacteria was used as the control sample for subsequent OxICAT-analysis. The resulting pellet was resuspended in 0.1% Triton (v/v) and passed five times through a 26-G needle in order to lyse the PLB-cells. Cell nuclei and large debris were removed by low-speed centrifugation (500 g, 5 min, 4 °C). The bacteria in the supernatant were recovered by following mid-speed centrifugation (3000 g, 10 min, 4 °C). The bacteria-containing pellet was rinsed once with 0.1% SDS (w/v) and pelleted (16.000 g, 5 min, 4 °C). This bacterial fraction was then immediately used for further analysis. The extracellular control obtained from the supernatant of the initial centrifugation (see above) was passed through a 26-G needle and treated with 0.1% Triton and SDS as well. This whole enrichment procedure was carried out in less than 45 min. Protein concentrations were determined using the Pierce™ BCA Protein Assay Kit (Thermo Scientific, Waltham, MA) and the relative bacterial protein content was monitored with Western Blot analysis using an antibody against GFP (1:4000, rabbit, Sigma-Aldrich), which was reactive to roGFP2-Orp1 expressed by *E. coli*.

### 2.5 SDS-PAGE and Western Blot

Protein samples were separated on 4–12% Bis-Tris Gels (NuPAGE™, Invitrogen, Carlsbad, CA) under reducing conditions (200 V, 40 min). For Western Blot analysis, proteins were transferred onto nitrocellulose membranes using the iBlot™ 2 Dry Blotting System (Invitrogen). Membranes were probed with antibodies against GFP (rabbit, 1:4000, Thermo Fisher Scientific). Proteins of interest were detected with fluorescent anti-rabbit antibody (goat, 1:10000, IRDye 680RD, LICOR, NE). Blots were imaged with an infrared imaging system (Odyssey Classic, LICOR, NE) using a three minute exposure time. Band intensity was determined using ImageJ [55].

### 2.6 OxICAT labeling of protein extracts

The OxICAT analysis was done according to the protocol of Lindemann and Leichert [37], [38]. Briefly, protein labeling was done using reagents provided by the Cleavable ICAT Method Development kit and Bulk kit (AB SCIEX, Framingham, MA). 100 µg of proteins were dissolved with one vial of the light-labeled ICAT predissolved in a mixture consisting of 80 µl DAB-buffer (6 M Urea, 200 mM Tris-HCl, 0.5% SDS, 10 mM EDTA, pH 8,5) and 20 µl acetonitrile (ACN), incubated for 2 h at 37 °C in the dark. Proteins from this solution were precipitated overnight at −20 °C with 80% acetone, rinsed twice with 1 ml of 80% acetone each and collected as pellet (4 °C, 16.000 g. 30 min). The pellet was dried at 37 °C for 5 min, dissolved in 80 µl DAB buffer containing 1 mM tris(2-carboxyethyl)phosphine hydrochloride (TCEP) and incubated for 10 min at 37 °C. This solution was mixed with one vial of the heavy-labeled ICAT resuspended in 20 µl ACN. This protein solution was incubated for 2 h at 37 °C in low-light conditions. Proteins were precipitated using acetone and rinsed as described. The resulting pellet was dissolved in 80 µl of Denaturing Buffer (50 mM Tris, 0.1% SDS) from the ICAT kits as mentioned above and mixed with 20 µl ACN and 100 µl of 0.125 µg/µl trypsin solution and incubated overnight at 37 °C. Subsequent peptide purification by cation exchange, avidin affinity chromatography and cleavage of the biotin-tag were performed according to the manufacturer´s instructions with the modification that the Affinity Buffer-Elute (30% ACN, 0.1% TFA (trifluoroacetic acid)) was freshly prepared at the day of experiment. Purified peptides were concentrated to dryness and dissolved in 0.1% TFA for LC-MS/MS analysis.

### 2.7 LC-MS/MS analysis

ICAT-labeled peptides were loaded onto a reverse phase nano-LC and detected by MS/MS in an LTQ Orbitrap Elite instrument (Thermo Fisher Scientific) as described [39]. In short, samples were loaded onto a C18 precolumn (100-µm × 2-mm Acclaim PepMap100, 5 µM, Thermo Fisher Scientific) with 0.1% TFA with 2.5% ACN (v/v) at a flow rate of 30 µl/min for 7 min. The peptides were then loaded onto the main column (75-µm × 50-cm Acclaim PepMap100 C18, 3 µm, 100-Å, Thermo Fisher Scientific) with 95% solvent A (0.1% formic acid (v/v)) and 5% solvent B (0.1% formic acid, 84% ACN (v/v)) at a flow rate of 0.4 µl/min. Subsequent elution was performed with a linear gradient of 5–40% B (120 min, 0.4 µl/min). The 6–20 most intense peaks were selected for MS/MS fragmentation (charge range +2 to +4, exclusion list size: 500, exclusion duration: 35 s, collision energy: 35 eV). The mass spectrometry proteomics raw data have been deposited to the ProteomeXchange Consortium [65] via the PRIDE partner repository with the dataset identifier PXD011386.

### 2.8 Quantification of cysteine oxidation using Maxquant

The MaxQuant software (version 1.5.1.0, DE) [12] was used to quantify the ICAT-labeled peptide thiols. For the search engine Andromeda, the *E. coli* K12 proteome database (taxonomy ID 83333) obtained from UniProt (4323 proteins, released September 2017, The UniProt Consortium, 2017) was used. For the Andromeda search, two miscleavages were allowed, Oxidation (M) was chosen as variable modification. The parent ion mass tolerance was set to 10 ppm, the fragment ion mass tolerance set to 0.5 Da. The oxidation of each identified peptide thiol and the relative oxidation change as compared to control samples were calculated from three biological replicates using the MaxQuant analysis. Identified peptides and their respective ICAT-quantification were assessed using the “peptides.txt” MaxQuant output file. In short, “peptides.txt” was imported into Excel 2016 (Microsoft). Then, values of each identified peptide from the column “Intensity H” was divided by the respective value from the column “Intensity” and multiplied by 100. This value equals the percentage of reversibly oxidized cysteine of the identified peptide and was used to determine cysteine oxidation in all samples. For further analysis, column bar diagram, volcano plot and heat map were generated using GraphPad Prism (version 6.01) and Microsoft Excel 2016. MaxQuant result files and the Excel result table can be accessed at the ProteomeXchange Consortium via the PRIDE partner repository under the dataset identifier PXD011386.

### 2.9 Bioinformatic data analysis

For the evaluation of conserved cysteines from OxICAT-identified proteins, the ConSurf server (www.consurf.tau.ac.il/2016/) was used [1]. HMMER [16] was used to obtain homologous sequences from the UNIREF-90 database (April, the 11th, 2018) with a E-value cutoff of 0.0001 and a maximum % identity between sequences of 95% and a respective minimum % identity of 35%. The top 150 sequences were retrieved for each protein and aligned using MAFFT-L-INS-I [34]. To calculate the relative surface accessibility of the identified cysteines, NetSurfP (www.cbs.dtu.dk/services/NetSurP/) was used [50].

### 2.10 Hydrogen peroxide growth inhibition assay

*E. coli* BW25113 as well as deletion strains used (Table 1) were obtained from the Keio collection (National Bio Resource Project, NIG, Japan) [2]. All strains were grown in LB-medium at 37 °C. At mid-logarithmic phase, bacteria cultures were split and diluted with LB-medium to a final OD_600_ of 0.03 and an H_2_O_2_ concentration of 2.5 mM or without H_2_O_2_ as a control. OD_600_ was measured every 30 min for up to 810 min at 37 °C. For quantification of the relative growth inhibition, the time for each strain to reach an OD_600_ of 0.2 was taken. For exact calculation, growth curves were fitted using third degree polynomials and the respective time was calculated from the fitted equation at OD_600_ of 0.2. The calculated values of each strain to reach an OD_600_ of 0.2 in the presence of hydrogen peroxide (T_H2O2_) was divided by the time of the respective strain to reach the same OD in the absence of H_2_O_2_ (T_control_). To enable comparison between strains, the relative growth inhibition was then normalized to the growth of WT bacteria, which was set to 1. For strains that did not reach an OD_600_ of 0.2 during the 810 min time course, T_H2O2_ was set to 840 min, as this would be the earliest time point they could have reached an OD of 0.2. For those strains, T_H2O2_ was used to determine the respective standard deviation and significance relative to the WT strain. Since the minimal relative growth inhibition calculated for these strains in this way was > 4, the relative growth inhibition was set to 4.

## 3 Results & discussion

### 3.1 Analysis and quantification of the *E. coli* thiol redox proteome in neutrophil cells by OxICAT

**Fig. 1:**
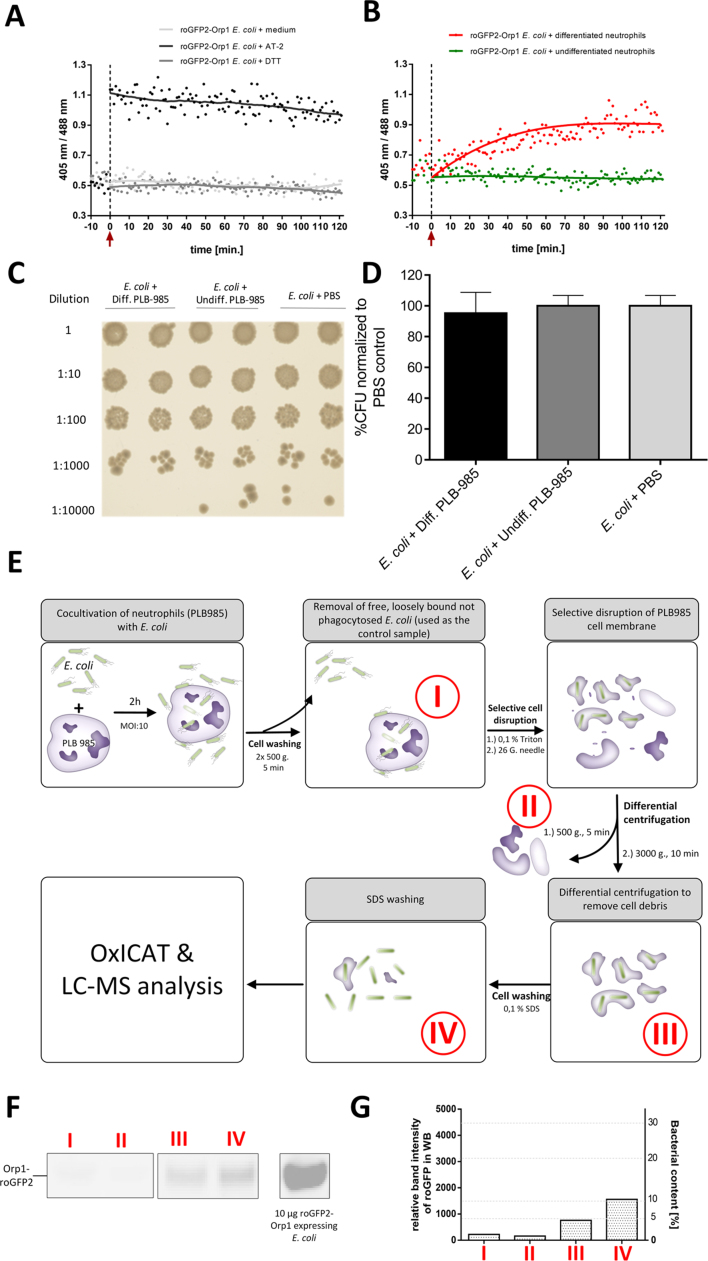
**Effects of phagocytosis by neutrophil-like PLB-985 cells on*****E. coli*****and enrichment of phagocytized bacteria A.** roGFP2-Orp1 expressing *E. coli* were mixed with 50 mM dithiothreitol (DTT), 1 mM aldrithiol-2 (AT-2) or **B.** neutrophil-like, differentiated PLB-985 cells (MOI (multiplicity of infection): ten *E. coli* to one PLB-985) as indicated by the red arrow. The redox state of roGFP2-orp1 was tracked for 120 min by measuring fluorescence intensities at 510 nm for both excitation wavelengths 488 nm and 405 nm. Once mixed with differentiated PLB cells, the redox-ratio of roGFP2-Orp1 expressing *E. coli* cells increased from 0.6 to 1.0 in a time-dependent manner and reached its maximum after approximately 80 min **C.** Serial dilutions of co-incubation assays (MOI: 2:10, 2 h of co-incubation) were plated on LB bacterial medium plates to determine killing by phagocytosis. **D.** No significant decrease in colony forming units (cfu) through action of differentiated PLB-985 was observed, when compared to undifferentiated PLB-985 cells or PBS, which served as control. **E.** Separation of phagocytized bacteria and non-phagocytized bacteria incubated with PLB-985 cells (MOI 10:1, I). Host cells were disrupted mechanically and centrifuged at 500 g to remove cell debris (II). Supernatant was centrifuged at 3000 g to collect the bacterial fraction (III), which was washed with 0.1% SDS (IV). Respective fractions taken for western blot are indicated by the roman numerals. **F. & G**. Western Blot of a normalized SDS-PAGE probed with α-GFP antibody (10 µg total protein per lane). Relative *E. coli* amount was determined by comparing the relative band intensity of roGFP2-Orp1 of each fraction to a known amount of lysate of *E. coli* from the same batch.

**Fig. 2:**
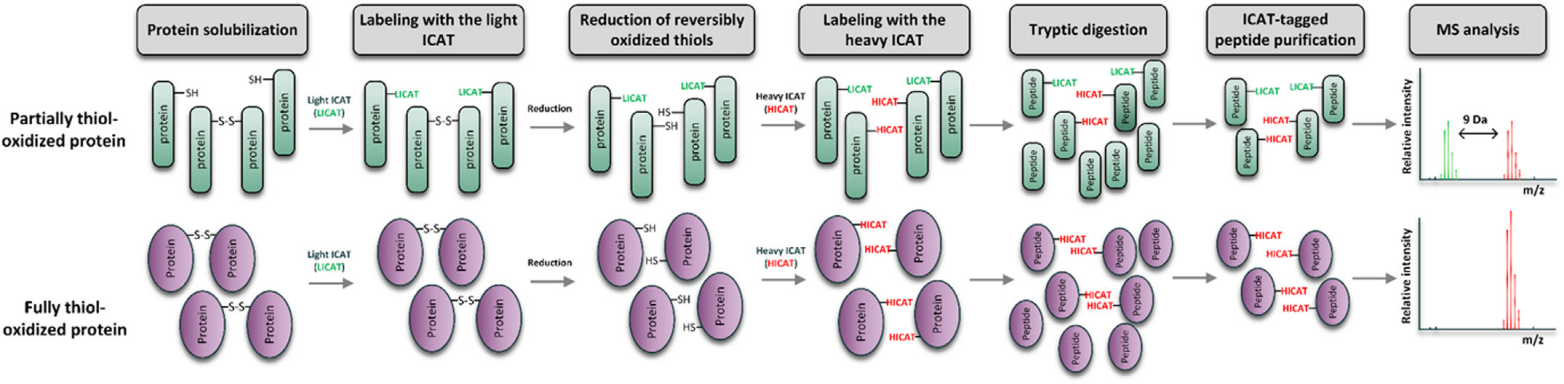
**Quantification of thiol-oxidation using OxICAT.** First, proteins of interest are solubilized and denatured, which allows the reaction of the isotopically light ^12^C-ICAT reagent (LICAT, green) with all free cysteines. Second, reversibly oxidized cysteines are reduced using Tris(2-carboxyethyl)phosphine and labeled with the isotopically heavy ^13^C-ICAT (HICAT, red). Then, the protein mixture is digested by trypsin and the ICAT-tagged peptides are purified using the biotin tag. Finally, the peptide mixture is analyzed using mass spectrometry. Partially thiol-oxidized proteins are labeled with both the LICAT and the HICAT. Fully oxidized thiol-oxidized proteins are labeled with HICAT only. The relative oxidation of a cysteine is reflected by the proportion of its respective LICAT- and HICAT-labeling.

Professional phagocytic immune cells produce a toxic mixture of different oxidative species to counteract against pathogenic intruders like bacteria. Recently we showed that the genetically encoded fluorescent redox probe roGFP2-Orp1 is promptly oxidized in bacteria that are phagocytized by neutrophils [14]. This suggested to us that *E. coli* is under significant oxidative stress once caught in the phagolysosome. The amino acid cysteine is a well-known target of oxidants produced in the phagolysosome of neutrophils [67], [69]. In our study, we were interested in analyzing the effect of oxidative stress on thiol redox proteome of *E. coli* having encountered host neutrophil phagocytosis. In order to find an optimal time point to harvest the cells for subsequent redox proteomic analysis, a 96-well format-based plate reader assay was used to monitor the oxidation state of roGFP2-Orp1 in the *E. coli* population over a time course of 2 h, while they were co-incubated with neutrophil-like PLB-985 cells. The oxidation state of roGFP2-Orp1 increased gradually, reaching a steady level after about 80 min of incubation and remained in an oxidized state until the end of the measurement (Fig. 1A and B). Nevertheless, bacteria were still fully viable when we plated serial dilutions of our co-incubation assay on LB medium after 2 h, demonstrating that probe oxidation is not caused by cell lysis (Fig. 1C and D). Thus, we decided to enrich intracellular bacteria after 2 h of co-incubation with PLB-985 cells, where the oxidation of roGFP2 in the cytoplasm of *E. coli* reached a steady level. For the subsequent redox proteomic

analysis, extracellular *E. coli* that were not phagocytized by PLB-985 were separated and served in this study as control. Due to the overwhelming amount of host cell proteins mixed with bacterial proteins [70], the development of a method to decrease the relative proportion of host cell proteins was necessary for the identification of bacterial proteins in LC-MS analysis. Several proteomic studies of bacterial pathogens upon interactions with host cells have been published. In these studies, differential centrifugation was widely used to enrich intracellular bacteria [41], [59], [70]. Based on those studies, we combined selective disruption of the host cell membrane, using 0.1% Triton X-100 and mechanical shearing, with subsequent differential centrifugation to enrich intracellular *E. coli* after interaction with neutrophils (Fig. 1E). We used roGFP2-Orp1 to assess the relative percentage of *E. coli* proteins in each fraction. roGFP2-Orp1 is a protein that was heterologously expressed in *E. coli* and could be quantified by Western Blot using an anti-GFP antibody. We then compared the relative band intensity of each fraction to *E. coli* lysates from the same batch. Based on this band intensity analysis (Fig. 1F and G), the fraction before enrichment contained less than 1.4% bacterial protein (fraction I). The final, enriched fraction contained approximately 10% of *E. coli* proteins (fraction IV). This enriched fraction, as well as extracellular *E. coli*, were then analyzed using the quantitative redox proteomic method OxICAT. In short, OxICAT is based on the differential labeling of protein thiols using an isotope-coded affinity tag reagent (ICAT). First, reduced protein thiols are labeled with the isotopically light ^12^C-ICAT. Second, reversibly oxidized thiols including disulfide bonds are reduced using Tris(2-carboxyethl)phosphine (TCEP) and labeled with the isotopically heavy ^13^C-ICAT. The oxidation state of a protein thiol is thus reflected by the proportion of light and heavy ICAT-labeled versions of the peptide containing the cysteine (Fig. 2) [37], [38].

**Fig. 3:**
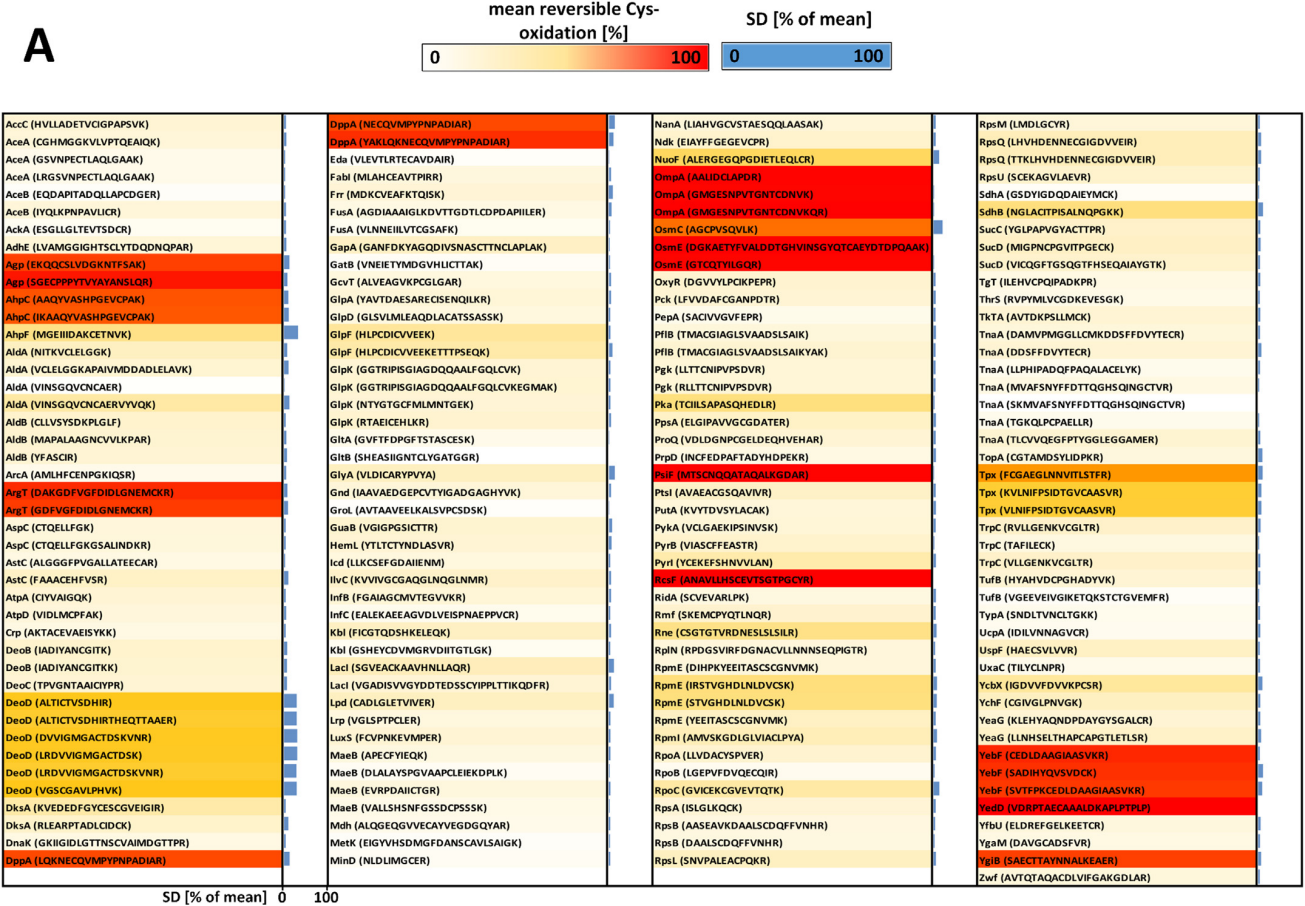

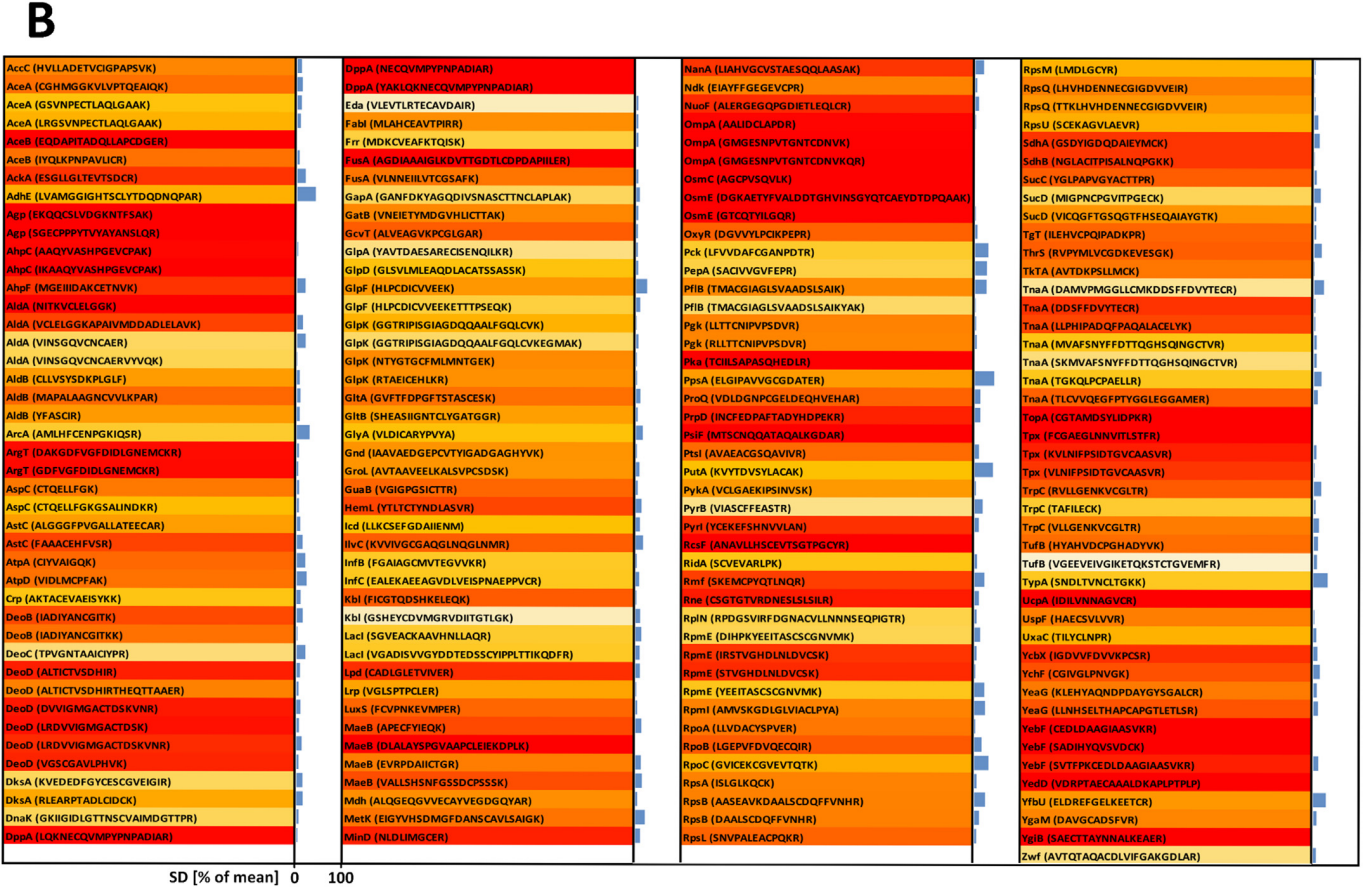
**Relative thiol oxidation of 173 identified cysteine-containing peptides in*****E. coli*****cells during phagocytosis.***E. coli* cells were incubated with neutrophils for 2 h. Extracellular *E. coli* cells were separated from the neutrophils and the intracellular *E. coli* cells enriched thereafter. Both the extracellular **(A)** and the intracellular **(B)***E. coli* cells were analyzed using OxICAT. The relative thiol oxidation of 173 matched cysteine residues were visualized using heat maps based on data shown in Supplementary Table 1. The white-yellow-red gradient denotes 0–100% oxidation. The standard deviation (SD) of three biological replicates is shown in blue.

In total, we could identify and quantify 173 matched cysteine containing peptides representing 117 proteins in each of our samples (Fig. 3, Suppl. Table 1). Given the fact that the *E. coli* genome encodes more than 4300 proteins, only a limited part of the *E. coli* proteome could be covered. In a quantitative condition-dependent *E. coli* proteome study, Schmidt et. al identified and quantified 2019 proteins from *E. coli* MG1655 grown in LB [54]. Thus, we cover only around 5.7% of the proteins that are known to be expressed in *E. coli* MG1655. The presence of contaminating host protein is probably, at least in part, reason for our limited coverage of *E. coli*'s proteome, and has been found to occur in other proteomic studies of bacteria-host interactions [70]. Additionally, the reactive group iodoacetamide of the ICAT reagent only reacts with reversibly oxidized thiols. It has been estimated that 5% of cellular protein cysteines are oxidized to sulfinic acids, an irreversibly oxidized form of thiol that does not react with iodoacetamide and could not be identified in the LC-MS analysis [25].

**Fig. 4:**
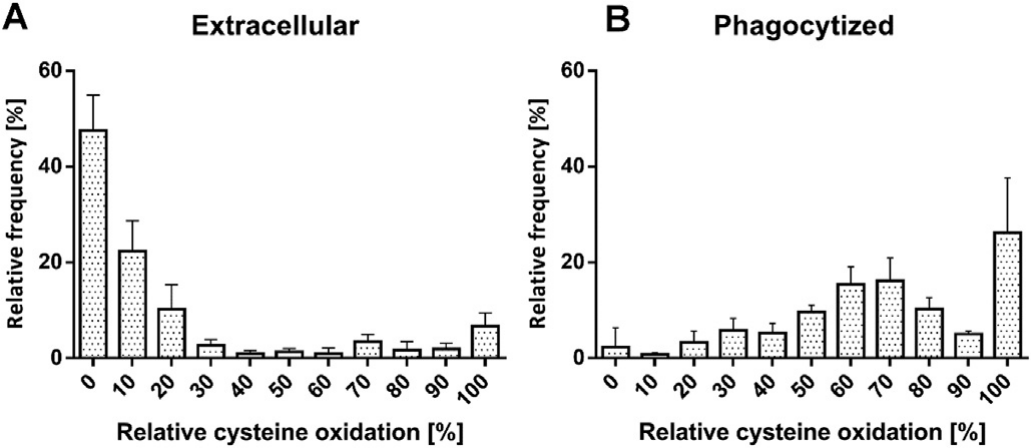
**Frequency distribution of the relative thiol oxidation of the identified cysteine-containing peptides in*****E. coli*****cells during phagocytosis**. *E. coli* cells were incubated with neutrophils for 2 h. Extracellular *E. coli* cells were separated from the neutrophils and the intracellular *E. coli* cells enriched thereafter. Both the extracellular **(A)** and the phagocytized **(B)***E. coli* cells were analyzed using OxICAT. The relative thiol oxidation of 173 matched cysteine residues were visualized using frequency distribution analysis.

**Fig. 5:**
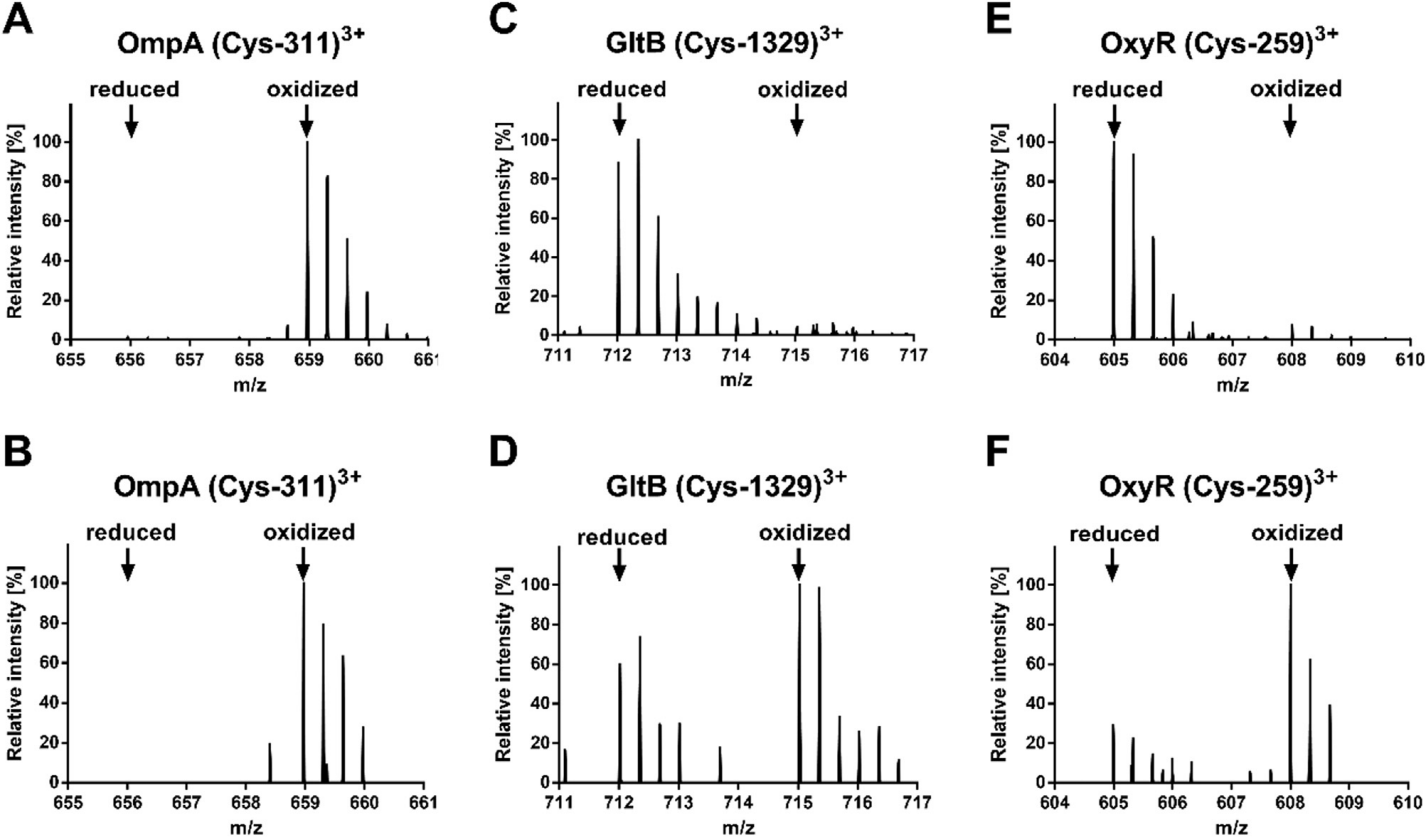
**MS-spectra of selected ICAT-labeled cytoplasmic and periplasmic protein thiols in extracellular and phagocytized *E*. *coli*.***E*. *coli* cells were co-incubated with a neutrophil like human cell line (PLB-985) at 37 °C for 2 h to allow phagocytosis. Protein thiols of both extracellular and phagocytized *E*. *coli* cells were enriched and differentially labeled with OxICAT. Mass signals of peptides labeled with the ^12^C-ICAT (reduced) and ^13^C-ICAT (oxidized) are shown. Cysteine 311 from the periplasmic protein OmpA was fully oxidized in both extracellular and phagocytized *E*. *coli* cells (**A, B**). Cysteine 1329 from the cytoplasmic protein GltB was predominantly reduced in extracellular *E*. *coli* cells and partially oxidized during phagocytosis (**C, D**). Cysteine 259 of OxyR was found more oxidized during phagocytosis as well (**E, F**).

In the control fraction, the vast majority, 135 peptides (78.0%) showed a thiol oxidation level of less than 20%, including 105 cysteine peptides (60.7%) with an oxidized fraction below 10% (Fig. 4). This indicates that most of the identified cysteine thiols were in their reduced state, suggesting that the cytoplasm of *E. coli* outside of the neutrophils is in an overall reducing state, as exemplified by the cytoplasmic glutamate synthase protein GltB (Fig. 5C). 23 peptides (12.7%) showed an oxidation level higher than 60% (Fig. 3, Fig. 4 and Supplementary Table 1). These highly thiol-oxidized proteins include periplasmic and outer membrane proteins such as OmpA (outer membrane protein A, up to 99.6% oxidized) and DppA (heme ABC transporter, up to 88.1% oxidized), which are known to harbor oxidized cysteine in the form of structural disulfide bonds and have already been reported as basal-level thiol-oxidized in *E. coli* (Fig. 5A) [37]. The resolving cysteine Cys-166 of AhpC (alkyl hydroperoxide reductase) was oxidized 78.6% in the control fraction containing extracellular *E. coli* (we were not able to identify the peptide containing the peroxidatic cysteine of AhpC in our experiments). This oxidation was more than 40% higher than the oxidized fraction of the resolving cysteine of AhpC we observed in *E. coli* cultured in minimal medium [37]. This increase in basal-level oxidation of this hydroperoxide detoxifying enzyme suggests that *E. coli* in close proximity to neutrophils already encounter a low-level of oxidative stress through oxidants such as H_2_O_2_ and monochloramine. These oxidants are produced in the phagosome and have been shown to be membrane permeable [23], [68].

In contrast to the overall low oxidation state of thiols in extracellular bacteria, *E. coli* that directly encountered neutrophil phagocytosis showed a thiol oxidation level of higher than 30% in 162 peptides (93.7%). These included 118 peptide cysteines (68.2%) oxidized even more than 60% (Fig. 3, Fig. 4 and Supplementary Table 1). Thus, the majority of identified cysteine residues in phagocytized bacteria were in an oxidized state.

### 3.2 Thiol-oxidized proteins from phagocytized *E. coli* are involved in protein and carbon metabolism

**Fig. 6:**
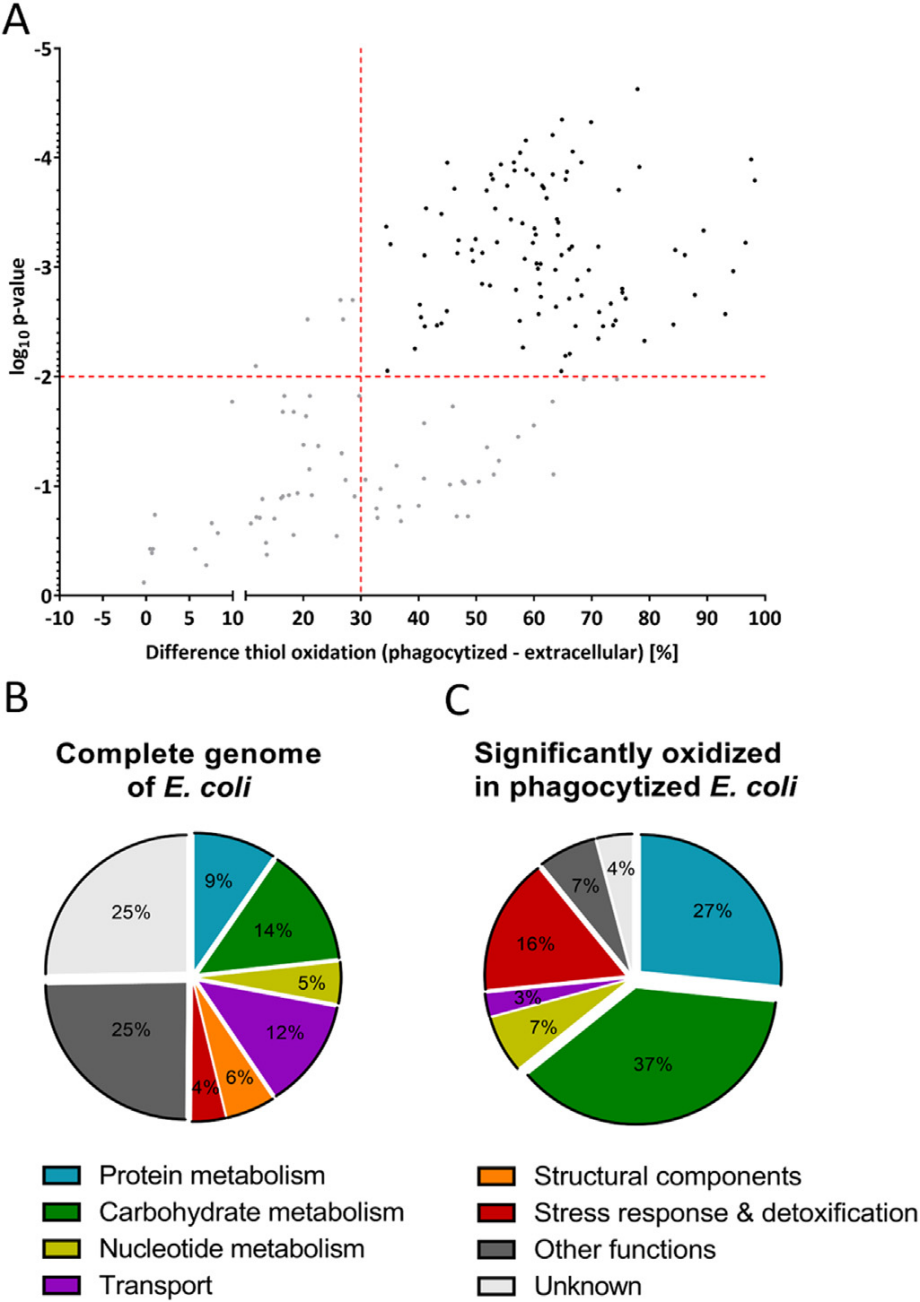
**Differentially oxidized protein cysteine residues of phagocytized*****E. coli*****and their functional classification. A.** A volcano plot shows the difference of thiol oxidation, as determined by OxICAT, between *E. coli* cells, which experienced host neutrophil phagocytosis to extracellular bacteria. Log_10_ of p-values from the Student´s t-distribution is plotted against the difference in thiol oxidation. The non-axial vertical line denotes an increase of 30% thiol oxidation while the non-axial horizontal line denotes a p-value of 0.01. Cysteine-containing peptides below these thresholds are shown as gray dots, peptides crossing the thresholds as black dots. **B.** All predicted proteins from *E. coli* K12 MG1655 (ecogene.org,16.05.2018) and their respective cellular functions. **C.** 76 Significantly thiol-oxidized proteins (difference > 30%; p > 0.01) and their respective cellular functions. Proteins involved in stress response, cell detoxification, carbohydrate and protein metabolism are overrepresented in our dataset.

**Fig. 7:**
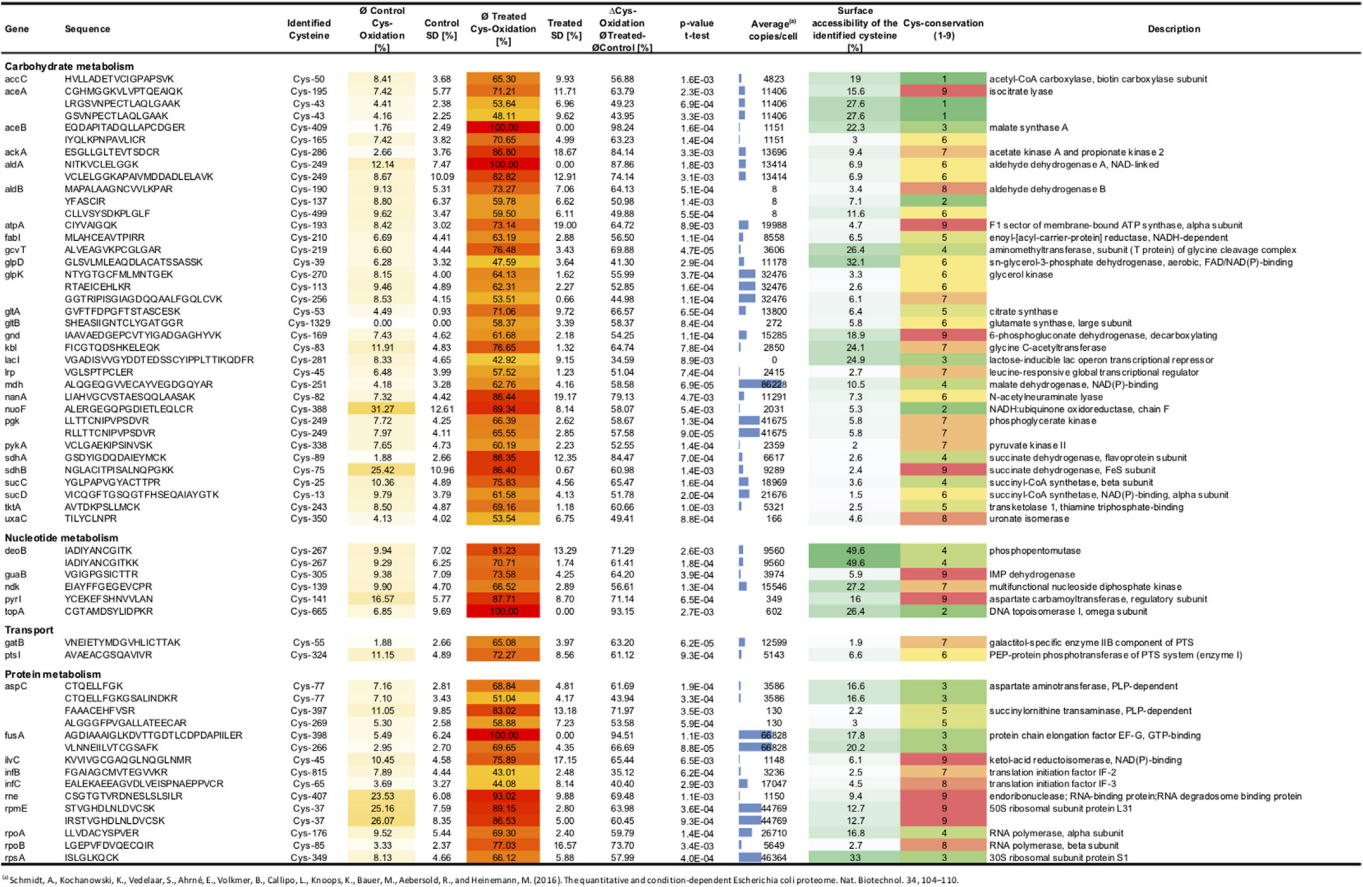

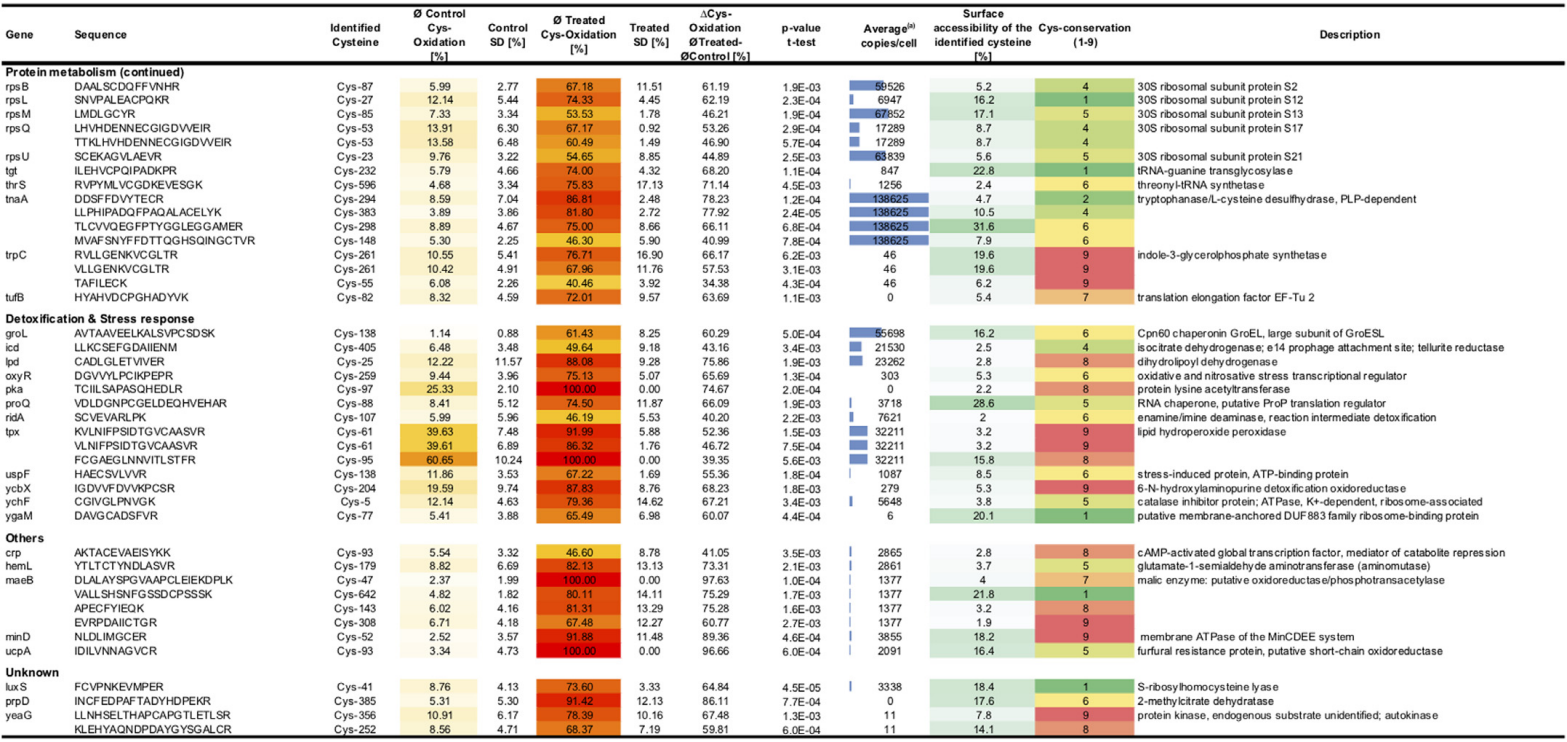
**Relative oxidation of OxICAT identified redox-active cysteine of*****E. coli*****having encountered neutrophil phagocytosis with a difference of > 30% thiol-oxidation after phagocytosis when compared to extracellular bacteria and p < 0.01**. Intracellular *E. coli* cells (Treated) were separated from extracellular bacteria (Control) and enriched according to Fig. 1E. The relative cysteine oxidation was determined using OxICAT. The average changes of thiol-oxidation after neutrophil phagocytosis (∆Cys-Oxidation) was determined by the difference between the treated (ØTreated) and the control (ØControl) samples. Protein function according to UniProt. The protein abundance was obtained from a protein expression database from *E. coli* MG1655 growing in LB medium ((a)Schmidt et. al 2016). The relative surface accessibility of identified cysteines was calculated using NetSurfP and the cysteine-conservation determined using Consurf. Values generated from three independent experiments.

To identify protein thiols that were affected by neutrophil phagocytosis, we compared the relative thiol oxidation state of phagocytized bacteria with that of extracellular bacteria. To select a set of significantly more oxidized cysteines in the phagocytized *E. coli*, Student´s *t*-tests were performed on the identified 173 cysteine-containing peptides. For the *t*-test, the percentage mean values of heavy-ICAT-labeled cysteine from each peptide were compared. Based on the mean values, significance in cysteine oxidation was determined between extracellular and phagocytized *E. coli*. The difference in thiol oxidation between those two samples as well as their respective p-values were then graphed onto a volcano plot. As thresholds, cysteine oxidation difference was set to 30% (non-axial vertical line) and the respective p-value to 0.01 (non-axial horizontal line). In this way, 102 peptide-containing cysteines representing 76 proteins were binned and showed a highly significant increase in thiol oxidation of more than 30% (Fig. 6, Fig. 7, Supplementary Table 2). The identified significantly oxidized proteins were predominantly from major metabolic pathways (Fig. 6B, C). In agreement with proteomic studies done with intracellular *Salmonella* species, most of the identified proteins were related to housekeeping functions [3], [59]. However, based on the *E. coli* genome, a noticeable high proportion of the identified proteins (16%) were involved in stress response and detoxification.

27% of the identified proteins were involved in protein synthesis. Amongst those, a significant number were ribosome associated proteins including Rne (ribonuclease E) and RpmE (50 S ribosomal protein L31), both showed oxidation at their conserved Zn-binding CXXC-motif. Further, essential components for the initiation of protein synthesis, including the translation initiation factors InfB (Cys-815) and InfC (Cys-65) were thiol-oxidized. Both RpmE and InfC have been shown to be oxidized in *E. coli* after HOCl-treatment [37]. Other oxidized proteins include FusA (elongation factor G), RpoA (DNA-directed RNA polymerase subunit alpha), RpsL (30S ribosomal protein S12) and RpsM (30S ribosomal protein S13). These were reported to be thiol-oxidized after allicin treatment, a thiol-oxidizing component from garlic that induces the oxidative and heat stress response in *E. coli* [47]. In addition, RpsM was identified as conserved S-thiolated protein in different Gram-positive bacteria under HOCl stress, such as *Corynebacterium glutamicum* and *Mycobacterium smegmatis* [7], [29]. The queuine tRNA-ribosyltransferase Tgt showed also increased oxidations under HOCl stress in *Staphylococcus aureus* [31]. The ketol-acid reductoisomerase IlvC, which was modified at the conserved Cys-45 after phagocytosis, is involved in the biosynthesis of isoleucine and valine. IlvC has been shown to harbor cysteine residues that were modified in *E. coli* under nitrosative stress, however the cysteines affected by NO• were not determined in that study [5]. In addition, TrpC (tryptophan biosynthesis protein TrpCF) was oxidized at Cys-261 and Cys-55. The significant amounts of identified proteins involved in translation and transcription suggests an inhibition of protein synthesis upon phagocytosis. Previous studies have shown that treatment with oxidants leads to the inhibition of protein synthesis in bacteria [19], [20], [40], [57]. Inhibition of protein synthesis upon phagocytosis might be used by host immune cells to stop cell division in bacteria. However, it has also been shown, that inhibition and reprogramming of transcription is used by bacteria to protect themselves against oxidative stress. Thus, it is possible that the inhibition of protein synthesis might be used initially by *E. coli* to respond to increased oxidative stress during the formation of the phagosolysosome [19], [40].

44% of all significantly oxidized proteins were from either carbohydrate or nucleotide metabolism. Oxidized conserved thiols were found in AceA (isocitrate lyase, Cys-195), Gnd (6-phosphogluconate dehydrogenase, Cys-169), AtpA (ATP synthase subunit alpha, Cys-193), GuaB (IMP dehydrogenase, Cys-305), Pyrl (aspartate transcarbamoylase, Cys-141) and SdhB (membrane-bound succinate dehydrogenase, Cys-75). Both AceA and GuaB were oxidized at their respective active site cysteines and hence most likely inactivated in phagocytized *E. coli*. GuaB belongs to the most conserved S-thiolated proteins in different Gram-positive bacteria [31]. AceA is used by *E. coli* to bypass the TCA cycle and enables the use of carbon substrates at the level of acetyl-CoA including fatty acids and alcohols [42]. AceA has been previously shown to be S-mycothiolated in *M. smegmatis* upon HOCl-treatment [29]. SdhB and the regulatory chain of PyrI were oxidized at their respective metal-binding sites. In addition to the implied inhibition of protein synthesis, we observed that neutrophil phagocytosis leads to the oxidation of proteins involved in major metabolic pathways and thus potentially their inactivation.

### 3.3 Neutrophil phagocytosis leads to thiol oxidation of antioxidant proteins and proteins involved in cell detoxification

Amongst the proteins in *E. coli* that were significantly thiol-oxidized after phagocytosis, some were known to be involved in the oxidative and heat shock stress response including Tpx (thiol peroxidase), RidA (enamine/imine deaminase), GroL (60 kDa chaperonin), ProQ (RNA chaperone) and OxyR (hydrogen peroxide-inducible genes activator). Tpx, a highly conserved thiol-specific peroxidase that preferentially catalyzes the reduction of alkyl hydroperoxides [24] was oxidized at both the peroxidatic cysteine (Cys-61) and the resolving cysteine (Cys-95) (Fig. 7, Supplementary Table 2). Tpx from different species were found more thiol-oxidized under HOCl-stress, including Tpx from *E. coli*, *M. smegmatis* and *S. aureus* [29], [31], [37]. RidA, that functions as a chaperone once N-chlorinated [46], was found 40% more oxidized at its conserved cysteine C107 after phagocytosis. Although the chaperone activity has been reported to be independent of C107, oxidation of this cysteine has been reported previously after peroxynitrite and allicin stress [39], [46], [47]. Other thiol-oxidized chaperones include GroL and ProQ. GroL promotes protein refolding under stress conditions and is known to be heat-responsive in *E. coli* [9]. Interestingly, GroL of the closely related *S.* Typhimurium was found induced during infection of macrophages [6]. ProQ was found to be involved in the DNA-damage response [61]. This points towards the possibility that both *E. coli* proteins involved in DNA-damage and protein-damage response are functionally occupied due to the oxidative environment present in the phagolysosome.

C259 from OxyR was also found oxidized (65.7%) after phagocytosis (Fig. 5E, F). OxyR is a master-regulator that controls the expression of antioxidant genes in response to both oxidative and nitrosative stress [26], [63], [71]. This is underlined by hypersensitivity of *oxyR* deletion mutants to hydrogen peroxide treatment and increased frequency of spontaneous mutagenesis [22], [63]. Redox signaling through OxyR is typically mediated by a disulfide formation between C199 and C208 [71]. Although we couldn´t identify peptides from OxyR containing either of the two cysteines, it has been shown that C259 forms a disulfide bond with C180. It was suggested that this disulfide bond might influence the regulatory mechanism of OxyR by facilitating disulfide formation of C199 and C208 [35].

### 3.4 Proteins modified upon neutrophil phagocytosis are needed by *E. coli* to overcome oxidative stress

**Fig. 8:**
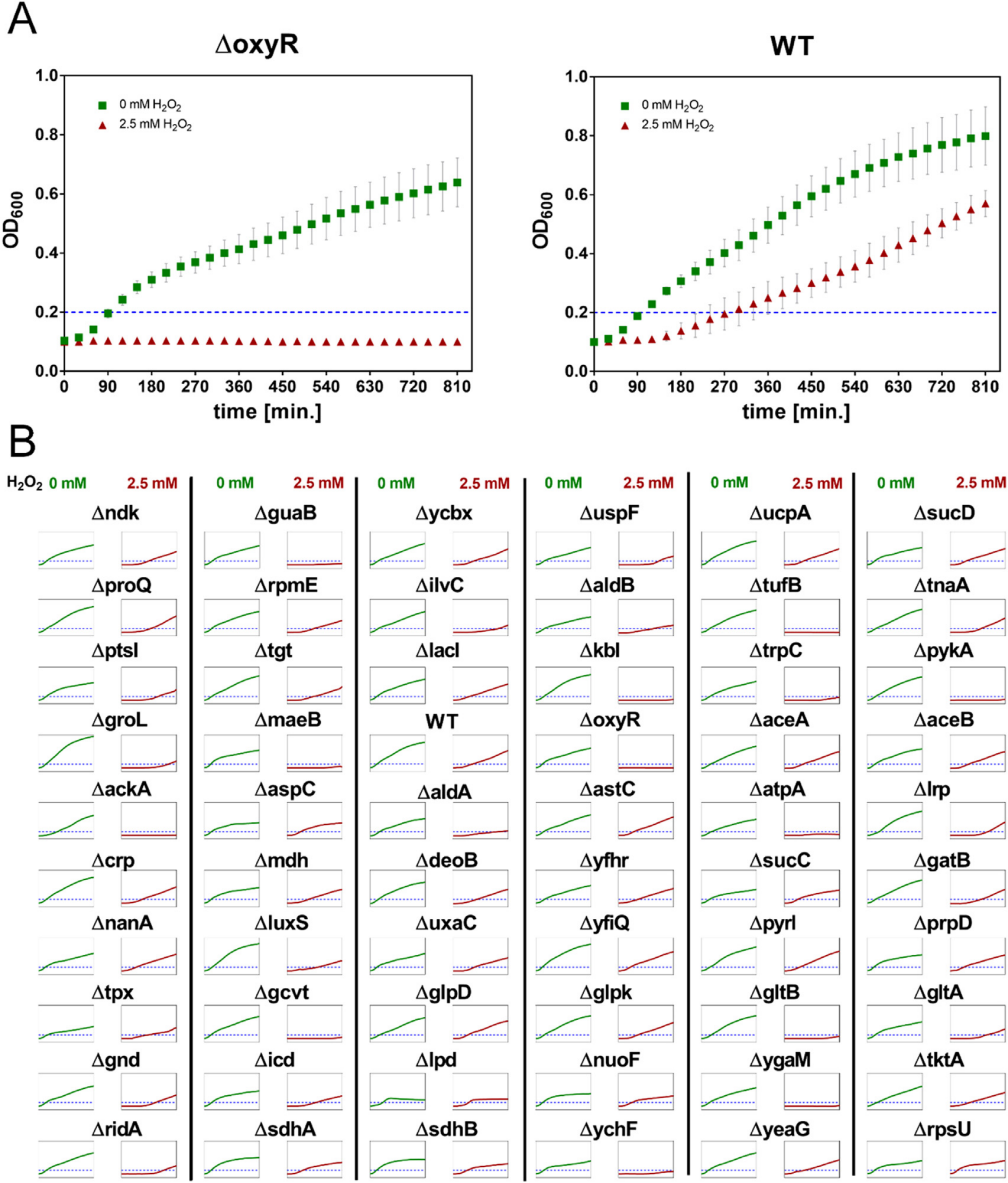
**Growth behaviour of single deletion*****E. coli*****mutants in the presence of oxidative stress. A.***E. coli* WT strain as well as the ∆*oxyR* deletion strain from the Keio collection were grown in LB-medium at 37 °C. At mid-logarithmic phase, cell cultures were diluted in LB-medium to a final OD_600_ of 0.03 and treated with 2.5 mM H_2_O_2_. The subsequent cell growth as reflected by OD_600_ is visualized. The time each culture needed to reach OD 0.2, as indicated by the blue non-axial dotted line, was used to quantify the H_2_O_2_ induced growth inhibition. For a detailed description of the calculation see part “Hydrogen peroxide growth inhibition assay” of the Materials and Methods section. **B.** Wild type (WT) and 59 deletion strains that lack non-essential proteins, which were shown to be significantly thiol-oxidized in the OxICAT-analysis, were tested for their respective H_2_O_2_ sensitivity.

**Fig. 9:**
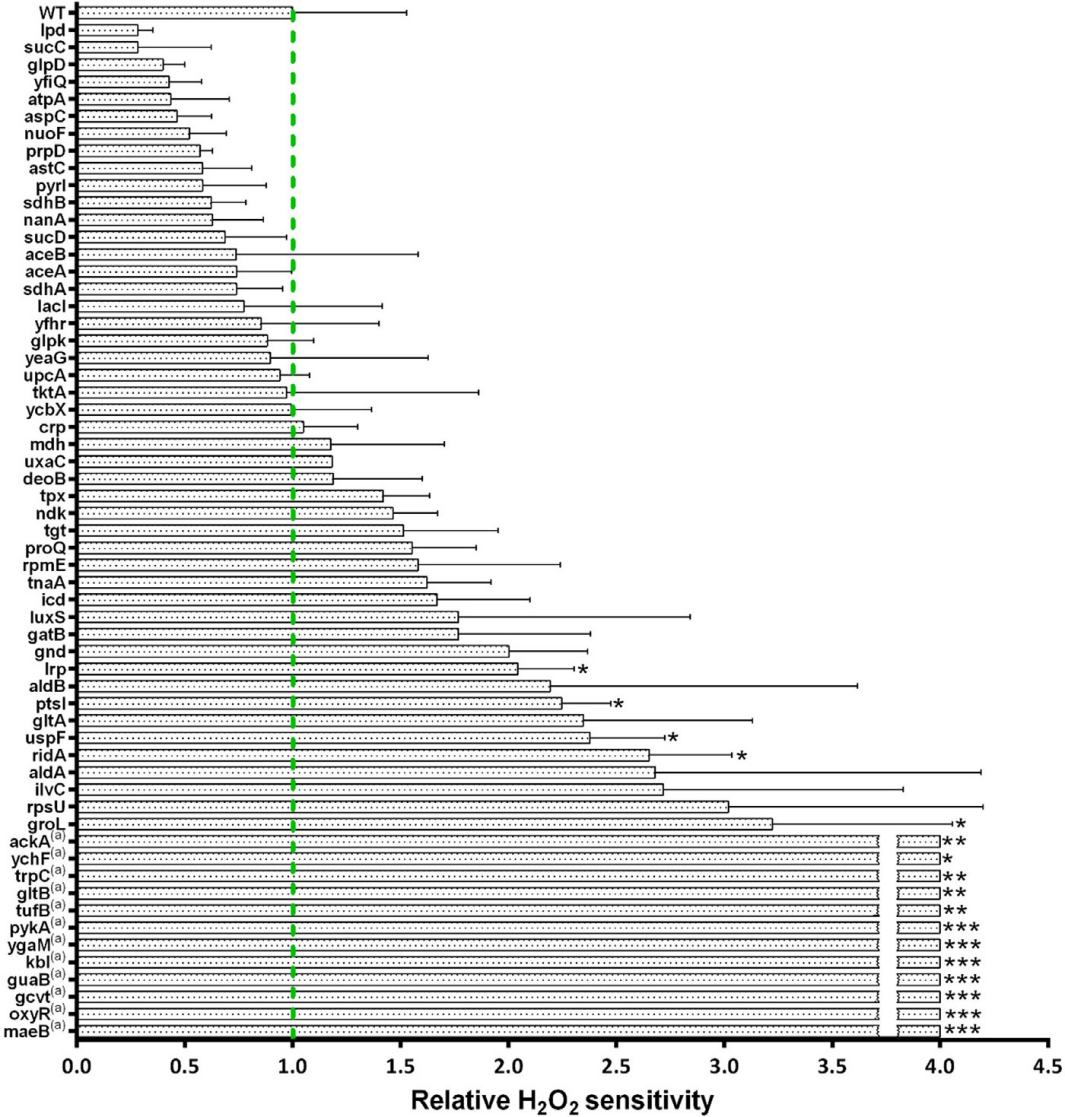
**H**_**2**_**O**_**2**_**sensitivity of 59*****E. coli*****deletion strains.***E. coli* wild type as well as 59 deletion strains chosen based on the OxICAT analysis were grown in LB medium at 37 °C. Mid-logarithmic cultures were split and grown in the absence and presence of 2.5 mM H_2_O_2_ in LB medium for 13.5 h. *E. coli* wild type needed approximately 5 h until it reached an OD_600_ = 0.2 in H_2_O_2_-containig media (see Fig. 8). The time needed for individual strains to reach an OD_600_ = 0.2 was used to calculate their relative H_2_O_2_ sensitivity in comparison to wild type. For a description of the calculation see part “Hydrogen peroxide growth inhibition assay” of the Materials and Methods section. Strains, that did not reach OD_600_ = 0.2 over 13.5 h were assigned the relative sensitivity value 4. All strains were normalized to the growth of WT *E. coli* cells (green dotted line). Significant difference compared to the WT cells was determined using Student´s *t*-test (*: 0.01 <p < 0.05, **: 0.001 <p < 0.01, ***: p < 0.001). Error bars show standard deviation.

One important weapon in the arsenal of a professional phagocytic cell is the production of different oxidative species. While HOCl is probably the most effective thiol oxidant released in the phagolysosme [14], other oxidants, such as hydrogen peroxide, are also present in high abundance and can lead to the damage of bacterial structures. To identify proteins with a potential antioxidant effect in *E. coli* during phagocytosis, we treated exponentially growing deletion strains with 2.5 mM H_2_O_2_ and measured the subsequent growth for 810 min (Fig. 8). From the 76 proteins significantly oxidized during phagocytosis, 17 were essential for *E. coli*. Thus, 59 deletion mutants lacking the non-essential genes were tested for H_2_O_2_-sensitivity. Several strains tested seemingly showed a lower relative H_2_O_2_ sensitivity (Fig. 9), however the differences were not significant and these strains typically already showed a growth defect under non-stress conditions (Fig. 8). On the other hand, 16 mutant strains showed significantly compromised growth upon treatment with H_2_O_2_ when compared to wild type (Fig. 8, Fig. 9). Amongst those, 11 strains did not reach an OD_600_ of 0.2 during the duration of our measurement (810 min). Thus, the respective genes deleted in theses 11 mutants are essential for efficient growth of *E. coli* in the presence of H_2_O_2_ (Fig. 9).

Similar to previous studies, the quorum-sensing mutants ∆*luxS* and ∆*tnaA* were not sensitive to H_2_O_2_ [36]. However, an *E. coli* mutant lacking the leucine-responsive regulatory protein Lrp has been shown to be more resistant to hydroperoxide stress [13]. In addition, overexpression of YchF, a highly conserved ATPase was shown to lead to H_2_O_2_ hypersensitivity in *E. coli* [66]. In agreement with previous studies, mutants lacking proteins that are important for the oxidative stress response, such as OxyR and RidA were significantly growth compromised [33], [46]. Furthermore, the heat shock responsive chaperone GroL, the malate dehydrogenase MaeB and the general stress responsive protein UspF were shown to be important for the growth of *E. coli* exposed to H_2_O_2_-stress [9], [48]. Similar to *Listeria monocytogenes*, the glutamate synthase GltB was shown in this study to be important for *E. coli* to respond to oxidative stress [30]. The hydrogen peroxide hypersensitivity of a *maeB* deletion mutant was reported for *S.* Typhimurium, and its sensitivity towards peroxynitrite was shown in *E. coli* [28], [38]. Both GltB and MaeB share the ability to reduce NADP^+^ to NADPH. NADPH is crucial for the functionality of cellular antioxidant enzymes including glutathione reductase and thioredoxin reductase [10], [11]. In addition, as suggested by Henard et. al, reduced generation of pyruvate (an effective scavenger of oxidants) might lead to the increased hydrogen peroxide sensitivity of a *maeB* deletion mutant [28], [49].

Some growth-inhibited mutants have not been reported to be responsive to oxidative stress. These include the metabolic enzymes TrpC (tryptophan biosynthesis protein) and Kbl (glycine C-acetyltransferase). Combined with our findings from the OxICAT analysis, our study highlighted the essentiality of some of those metabolic enzymes for the survival of *E. coli* under oxidative stress.

## 4 Conclusion

In humans, neutrophils are the most abundant circulating leukocytes. They are immediately recruited to sites of inflammation to eliminate invading pathogens. Pathogens, such as bacteria, are then engulfed and trapped in phagosomes once they encounter neutrophils. In the phagosomes, bacteria are attacked by a complex mixture of different oxidants produced by the neutrophils. We studied the effects of neutrophil phagocytosis on the thiol proteome of bacteria. Based on our data, we conclude that neutrophil phagocytosis leads to an overall break-down of the *E. coli* protein thiol homeostasis. Amongst the proteins we identified were numerous proteins needed by *E. coli* to survive oxidative stress. Thus, our study suggests that a systemic oxidation of protein thiols might be a general antimicrobial mechanism that neutrophils have at their disposal to counteract invading bacteria.
